# Transcription of the Extensively Fragmented Mitochondrial Genomes of Human Lice

**DOI:** 10.3390/biology15040296

**Published:** 2026-02-08

**Authors:** Emily Dunn, Renfu Shao

**Affiliations:** 1 Centre for Bioinnovation, University of the Sunshine Coast, 90 Sippy Downs Drive, Sippy Downs, QLD 4556, Australia; 2 School of Science, Technology and Engineering, University of the Sunshine Coast, 90 Sippy Downs Drive, Sippy Downs, QLD 4556, Australia

**Keywords:** fragmented mitochondrial genome, gene expression, minichromosome expression, mitochondrial genome transcription, human lice

## Abstract

The mitochondrial (mt) genomes of animals, including humans, are typically a single circular chromosome containing all mt genes. In several animal lineages, however, mt genomes have become fragmented, with genes distributed on multiple minichromosomes. How fragmented mt genomes are transcribed is still poorly understood. In this study, we investigated the transcription of the extensively fragmented mt genomes of the human head louse (*Pediculus humanus capitis*) and the human body louse (*Pediculus humanus corporis*). RNA-seq reads of both subspecies were retrieved from the NCBI Sequence Read Archive database and mapped to their mt genomes. The transcription level of each mt gene, minichromosome, motif, coding region and non-coding region, measured by RPKM (Reads Per Kilobase of transcript per Million mapped reads), TPM (Transcripts Per Million) or read coverage, was analysed statistically. In both subspecies, mt minichromosomes were transcribed entirely, with coding regions transcribed at much higher levels than non-coding regions. The 37 mt genes are transcribed unevenly, with *rrnL*, *cox1*, *cox2*, *cox3* and *atp6* transcribed at significantly higher levels than several other genes. Many transcription events terminate near a GC-rich motif in the non-coding regions; however, some transcription events pass this motif, leading to the transcription of entire non-coding regions. Despite the drastic difference in mt genome organisation, the human lice share several transcriptional features with humans, but also have unique features related to their fragmented mt genome organisation. The current study represents the first effort into the transcription of fragmented mt genomes. As more RNA-seq data become available, further studies on other animals with fragmented mt genomes are necessary to fully understand how genome fragmentation affects transcription.

## Simple Summary

Genome fragmentation is a drastic, large-scale mutation. How genome fragmentation affects gene and chromosome transcription is still poorly understood. In this study, we analysed the RNA-seq data of human lice to understand how their extensively fragmented mitochondrial genomes are transcribed. We find their mitochondrial minichromosomes are transcribed entirely, with genes transcribed at much higher levels than non-coding regions. The 37 mitochondrial genes of human lice are transcribed unevenly—several genes are transcribed at significantly higher levels than other genes. Many transcription events terminate near a highly conserved GC-rich motif in the non-coding regions; however, some transcription events can pass this motif, leading to the transcription of entire non-coding regions. Despite the drastic difference in mitochondrial genome organisation, the human lice share several transcriptional features with humans, but also have unique features related to their fragmented mitochondrial genome organisation. The current study represents the first major effort in the transcription of fragmented genomes and will serve as a stepping stone for further studies on other animals and eukaryotes with fragmented genomes.

## 1. Introduction

Mitochondria are organelles in the cells of eukaryotes that produce cellular energy [1,2]. Mitochondria contain their own genome, separate from the nuclear genome; however, the function of mitochondria relies on proteins coded by both mitochondrial (mt) and nuclear genomes [2,3]. Typically, animal mt genomes contain a single, circular chromosome of around 15,000 base pairs (bp) in size [4]. In the past decade, fragmented mt genomes have been found in several animal lineages, with mt genes distributed on two chromosomes or multiple minichromosomes. These animal lineages include sponges [5,6], cnidarians [7,8], rotifers [9], mesozoans [10], nematodes [11], book lice [12,13], and parasitic lice [14,15]. Fragmented mt genomes in parasitic lice were first discovered in *Pediculus humanus corporis* (human body louse) and later in the closely related *Pediculus humanus capitis* (human head louse) and many other species [14,15,16,17,18,19,20,21,22]. The mt genomes of both *P. humanus* subspecies contain 20 minichromosomes, each with one to three genes and a large non-coding region [14,15].

Transcription of the typical single-chromosome animal mt genomes has been studied in detail in humans [3,23,24]. Transcription of the human mt genome involves three main steps: initiation, elongation and termination [1,3,23,24]. Transcription begins at initiation sites after RNA polymerase binds to promoter regions on the mt genome [1,3,23,24]. Once initiated, transcription proceeds along either the partial or entire mtDNA strand, producing a polycistronic RNA. Transcription can be terminated by a protein called mitochondrial transcription terminator (mtTERM), which binds to the termination site, bends the DNA helix and stops transcription. The polycistronic RNAs are then processed to produce individual mRNAs, rRNAs and tRNAs. Different parts of the human mt genome tend to be transcribed unevenly [2,25,26]. Functional genes tend to be transcribed at higher levels than non-coding regions, and within the coding regions, rRNA genes are transcribed at higher levels than protein-coding genes and tRNA genes [2,25,26].

Very little is known currently about how mt genome fragmentation may affect transcription [14]. In human lice, an AT-rich motif and a GC-rich motif in the non-coding region are known to be highly conserved among all mt minichromosomes in both *Pediculus* subspecies and are therefore hypothesised to be involved in transcription initiation and termination [14,15]. However, this has not been investigated. The non-coding regions account for more than half of the mt genome size of human lice; whether these non-coding regions are transcribed partially or entirely is unknown. It is also unknown whether the 20 mt minichromosomes are transcribed on their own or in a coordinated manner [15]. In this study, we investigated the transcription of the extensively fragmented mt genomes of human lice by exploring the RNA-seq data available. RNA-seq is a powerful tool for investigating transcriptomes [2,27]. RNA-seq technology has the advantages of high-throughput and single-base resolution; genome transcription can be studied by mapping RNA-seq reads to reference genomes [27]. RNA-seq read depth or coverage can be used to determine the transcribed regions of the genome and compare the transcription levels between different genes or between different samples [2,27]. The RNA-seq data of human lice, in particular, are valuable for understanding how fragmented animal mt genomes are transcribed. In this study, we focus on three questions: (1) Where are the transcription initiation and termination sites on each mt minichromosome? (2) Are the non-coding regions of mt minichromosomes transcribed? And (3) are different mt minichromosomes transcribed on their own or in a coordinated manner?

## 2. Materials and Methods

### 2.1. Retrieval of RNA-Seq Data

RNA-seq data available for the human head louse *P. humanus capitis* and the body louse *P. humanus corporis* were retrieved from the NCBI Sequence Read Archive (SRA) database (https://www.ncbi.nlm.nih.gov/sra, accessed on 15 May 2024). Six data sets were available for *P. humanus capitis*, and two data sets were available for *P. humanus corporis* (Table S1). The RNA-seq data sets were retrieved from the SRA database using the Galaxy Australia platform (https://usegalaxy.org.au, accessed on 15 May 2024). Four of the six head louse data sets were produced with the oligo-dT selection method (Table S1), which selectively amplifies messenger RNA molecules [28]. The other two data sets of *P. humanus capitis* and the two data sets of *P. humanus corporis* were produced with a random selection method, which does not selectively amplify any specific types of RNA molecules [28]. A summary of the genes, minichromosomes and long non-coding regions investigated is provided in Table S2.

### 2.2. Quality Assessment and Analysis of RNA-Seq Data

The RNA-seq data sets of *P. humanus capitis* and *P. humanus corporis* were evaluated using the FASTQ Quality Control tools in Galaxy Australia. Trimmomatic tools (HEADCROP and AVGQUAL) were used to remove low-quality reads. The resulting RNA-seq data sets were mapped to the reference mt genome sequences of *P. humanus capitis* and *P. humanus corporis* in Geneious Prime (Version 2024.0.2) (http://www.geneious.com, accessed on 15 May 2024). The annotated mt genomes of *P. humanus capitis* and *P. humanus corporis* were retrieved from GenBank (Accession numbers: FJ499473–FJ499490; and JX080388–JX080477). Both *P. humanus* subspecies have two pairs of minichromosomes with similar gene content. The first pair is *trnL*_1_*-rrnS-trnC* and *trnL*_2_*-rrnS-trnC*, and the second pair is *trnL*_1_*-rrnL* and *trnL*_2_*-rrnL*. In both pairs, *trnL*_1_ and *trnL*_2_ differ by only a single nucleotide [14,15]. Each pair was treated as a single minichromosome for mapping purposes in the current study to ensure accurate measurement of the read coverage of each gene in these minichromosomes. As the *trnL* gene upstream of *rrnS* was mostly *trnL*_1_ (93.3%) [18], the coverage of this *trnL* was assigned to *trnL*_1_. Conversely, the *trnL* gene upstream of *rrnL* was mostly *trnL*_2_ (56.5%) [18], so the coverage of this *trnL* was assigned to *trnL*_2_. The annotated mt gene sequences of each *P. humanus* subspecies were concatenated as one sequence for mapping with the RNA-seq reads, while the non-coding regions (including a conserved GC-rich motif and a conserved AT-rich motif) available for each subspecies were mapped individually, due to the fact that they have high similarity to one another [15]. Full-length non-coding region sequences were available for the *cox1* minichromosome and *trnK-nad4* minichromosome of *P. humanus capitis*, and available for 12 minichromosomes of *P. humanus corporis*: *atp8-atp6*, *cob*, *cox1*, *trnY-cox2*, *cox3-trnA*, *nad1-trnQ*, *trnP-nad2-trnI*, *trnR-nad3*, *trnK-nad4*, *nad5*, *trnL*_1_*-rrnS-trnC* and *trnL*_2_*-rrnL* (Table S2). The RNA-seq data sets of each subspecies were mapped respectively to the concatenated mitochondrial minichromosome sequences and each available full-length non-coding region sequence, using Geneious Prime. As the number of reads varied among different RNA-seq data sets (Table S1), to make the transcription level measurement comparable among these data sets, we normalised the read coverage for each mt gene, minichromosome and motif using RPKM (Reads Per Kilobase of transcript per Million mapped reads) and TPM (Transcripts Per Million), calculated in Geneious Prime (Table S3). For RPKM and TPM calculation in Geneious Prime, *Count as multiple full matches* was selected for the *Ambiguous Mapper Reads* setting. RPKM was used for comparison between different genes, minichromosomes, or motifs within the same data set; TPM was used for comparison between the same genes or minichromosomes of different data sets; the RPKM of the GC-rich motif was compared with that of the adjacent 50 bp downstream to identify transcription termination sites; and the RPKM of the AT-rich motif was compared with that of the adjacent 50 bp upstream to identify transcription initiation sites (Table S4). The RPKM data of coding region and noncoding region of each minichromosome were calculated in two different mapping files and were not suitable for direct comparison. We thus used the mean read coverage, calculated in Geneious Prime, as an alternative for transcription-level comparison between the coding region and the non-coding region of each mt minichromosome (Table S5).

### 2.3. Statistical Analysis

The RPKM, TPM and mean coverage data (Tables S3–S5) were imported into IBM SPSS Statistics (Version 29) for statistical analysis of transcription levels. To determine the appropriate statistical tests to use for comparison, normality tests were performed first for the transcription level data of mt genes, minichromosomes, sequence motifs, coding regions, and non-coding regions, many of which failed the test of normality (Table S6). Thus, non-parametric statistical tests, including the Wilcoxon signed-rank test (for comparison between two data groups) and the Friedman test (for comparison among three or more data groups), were used in the current study. The Wilcoxon signed-rank test and the Friedman test were selected because they were paired tests and the data groups of comparison in the current study were all paired, e.g., different genes, minichromosomes, motifs or regions within the same RNA-seq data set, or the same gene between different RNA-seq data sets. The statistical significance level was set at *p* < 0.05. The transcription levels between the coding and non-coding regions of *P. humanus capitis* were compared for the *trnK-nad4* minichromosome. The *cox1* minichromosome of *P. humanus capitis* was excluded from this comparison due to its biased, extremely high read coverages in a few segments of the non-coding region. For *P. humanus corporis*, the transcription levels between the coding and non-coding regions were compared for 11 of the 12 available minichromosomes (*atp8-atp6*, *cob*, *cox1*, *trnY-cox2*, *cox3-trnA*, *trnP-nad2-trnI*, *trnR-nad3*, *trnK-nad4*, *nad5*, *trnL*_1_*-rrnS-trnC* and *trnL*_2_*-rrnL*). The *nad1-trnQ* minichromosome of *P. humanus corporis* was excluded from this comparison due to its biased, extremely high read coverages in a few segments of the non-coding region.

## 3. Results

### 3.1. Mitochondrial Gene Transcription Level Differs Between Human Head Lice and Body Lice, Between Different Development Stages, and Between Different Organs

Two head louse data sets of males (SRR24460203, SRR24460204) are significantly higher than a body louse data set of male (SRR24460210) in mt gene transcription level (Table 1). All three data sets were from male non-specified tissues and generated with the same sequencing strategy (Table 1 and Table S1). There is no significant difference between the two head louse data sets of males (SRR24460203, SRR24460204), nor between the two body louse data sets of males (SRR24460210, SRR24460211) (Table 1). The two body louse data sets of males (SRR24460210, SRR24460211) are significantly lower in mt gene transcription level than the 5-day-old-accessory-gland data set of the female head louse (SRR13528754) (Table 1). These differences, however, need to be interpreted with caution as SRR24460210 and SRR24460211 differ from SRR13528754 not only in gender, age and tissue type but also in sequencing strategy (Table S1). The 0-day-old-ovary data set of female head louse (SRR13528753) is significantly higher in mt gene transcription level than the 5-day-old-ovary data set of the female head louse (SRR13528752); the same is true for the 0-day-old-accessory-gland data set of the female head louse (SRR13528755) relative to the 5-day-old-accessory-gland data set of the female head louse (SRR13528754) (Table 1). These four data sets (SRR13528752–SRR13528755) were generated with the same sequencing strategy (Table S1). There is no significant difference between the 0-day-old-accessory-gland data set (SRR13528755) and the 0-day-old-ovary data set (SRR13528753) in mt gene transcription level; there is no significant difference either between the 5-day-old-accessory-gland data set (SRR13528754) and the 5-day-old-ovary data set (SRR13528752) in mt gene transcription level (Table 1). The head louse female 0-day-old-ovary data set (SRR13528753) was significantly higher in mt gene transcription level than the two head louse data sets of males (SRR24460203, SRR24460204, non-specified tissues) (Table 1). These differences, again, need to be interpreted with caution, as SRR13528753 differs from SRR24460203 and SRR24460204 not only in gender, age and tissue type but also in sequencing strategy (Table S1). On the other hand, these two head louse data sets of males (SRR24460203, SRR24460204, non-specified tissues) differ from the head louse female 5-day-old-ovary data set (SRR13528752), and the 5-day-old-accessory-gland data set (SRR13528754), and the 0-day-old-accessory-gland data set (SRR13528755) in three factors (gender, age and tissue type) (Table S1), but have no significant difference from these data sets in mt gene transcription level (Table 1).

### 3.2. Mitochondrial Minichromosomes Are Transcribed Entirely, with Coding Regions Transcribed at Significantly Higher Levels than Non-Coding Regions

**Figure 1:**
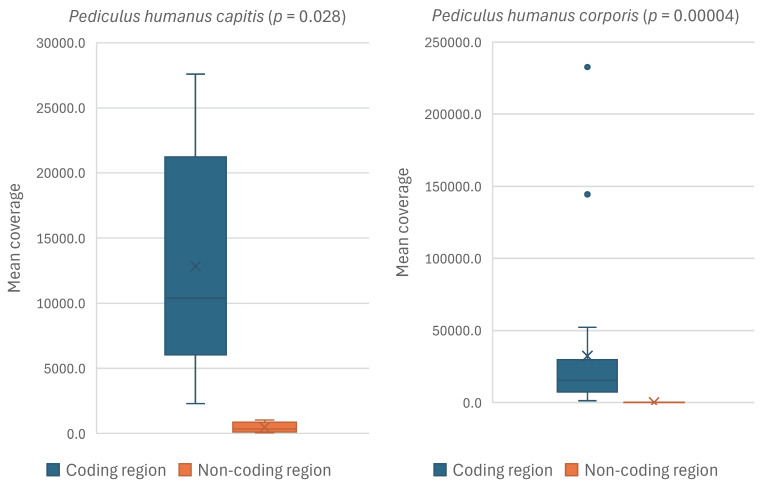
Comparison of transcription level between the coding and non-coding regions of each mitochondrial minichromosome of the human head louse *Pediculus humanus capitis* (**left**) (*cox1* and *trnK-nad4* minichromosomes available for comparison), and the human body louse *P. humanus corporis* (**right**) (*atp8-atp6*, *cob*, *cox1*, *trnY-cox2*, *cox3-trnA*, *nad1-trnQ*, *trnP-nad2-trnI*, *trnR-nad3*, *trnK-nad4*, *nad5*, *trnL*_1_*-rrnS-trnC* and *trnL*_2_*-rrnL* minichromosomes available for comparison), using the Wilcoxon signed-rank test (Tables S7 and S8). The *p*-value is displayed for each subspecies.

Twelve mt minichromosomes of *P. humanus corporis* (*atp8-atp6*, *cob*, *cox1*, *trnY-cox2*, *cox3-trnA*, *nad1-trnQ*, *trnP-nad2-trnI*, *trnR-nad3*, *trnK-nad4*, *nad5*, *trnL*_1_*-rrnS-trnC* and *trnL*_2_*-rrnL*) and two mt minichromosomes of *P. humanus capitis* (*cox1* and *trnK-nad4*) have been sequenced entirely for both coding and non-coding regions [14,15]. The *cox1* minichromosome of *P. humanus capitis* and the *nad1-trnQ* minichromosome of *P. humanus corporis* were excluded from the comparison of transcription level due to biased, extremely high read coverages in a few segments of their non-coding regions. RNA-seq read mapping showed that all the mt minichromosomes of *P. humanus corporis* and *P. humanus capitis* were transcribed entirely except for a few short sections (<50 bp) that were not transcribed in a few RNA-seq data sets. The mean read coverages of the coding region and non-coding region of each mt minichromosome were compared using the Wilcoxon signed rank test. In both *P. humanus corporis* and *P. humanus capitis*, the coding region was transcribed at substantially higher levels than the non-coding region of the same minichromosome, with *p* = 0.028 for *P. humanus capitis* and *p* = 0.00004 for *P. humanus corporis* (Figure 1; Tables S7 and S8).

### 3.3. Transcription Level Varies Among Mitochondrial Minichromosomes

**Figure 2:**
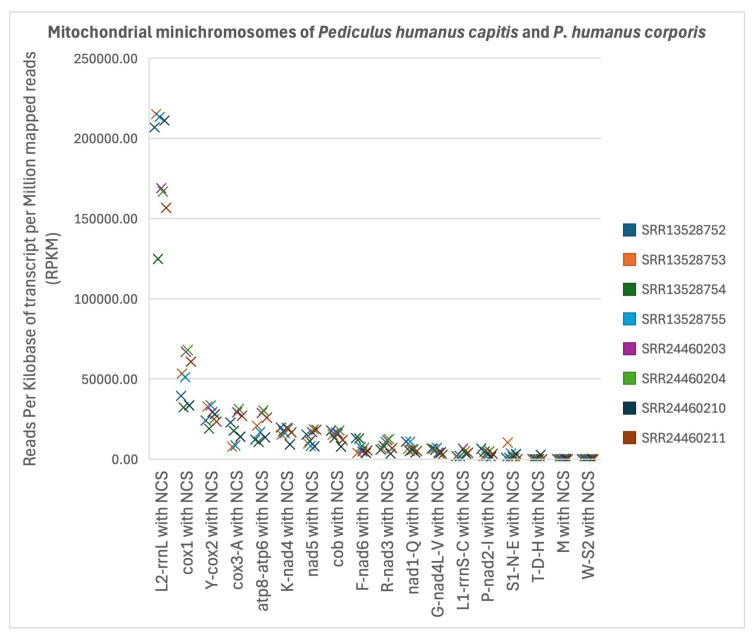
Comparison of transcription level among mitochondrial minichromosomes of both the human head louse, *Pediculus humanus capitis*, and the human body louse, *P. humanus corporis* using the Friedman test; *p*-values are provided in Table S9 for each pair of mitochondrial minichromosomes. Details of the eight RNA-seq data sets are in Table S1.

Pair-wise comparison among mt minichromosomes using the Friedman test showed that minichromosomes were not transcribed at the same level. When all the data sets of *P. humanus capitis* and *P. humanus corporis* were combined and analysed as a whole, the *trnL*_2_*-rrnL* minichromosome and *cox1* minichromosome were significantly higher in transcription level than seven other minichromosomes: *trnG-nad4L-trnV* (*p* < 0.05), *trnL*_1_*-rrnS-trnC* (*p* < 0.005), *trnM* (*p* < 0.00001), *trnP-nad2-trnI* (*p* < 0.005), *trnS*_1_*-trnN-trnE* (*p* < 0.005), *trnT-trnD-trnH* (*p* < 0.00001), and *trnW-trnS*_2_ (*p* < 0.00001) (Figure 2, Table S9). The *trnY-cox2* minichromosome was significantly higher in transcription level than six other minichromosomes: *trnL*_1_*-rrnS-trnC* (*p* < 0.05), *trnM* (*p* < 0.001), *trnP-nad2-trnI* (*p* < 0.05), *trnS*_1_*-trnN-trnE* (*p* < 0.05), *trnT-trnD-trnH* (*p* < 0.001), and *trnW-trnS*_2_ (*p* < 0.0001) (Figure 2, Table S9). The *atp8-atp6* minichromosome, *cob* minichromosome, *cox3-trnA* minichromosome, and *trnK-nad4* minichromosome were significantly higher in transcription level than three other minichromosomes: *trnM* (*p* < 0.05), *trnT-trnD-trnH* (*p* < 0.05), and *trnW-trnS*_2_ (*p* < 0.01) (Figure 2, Table S9). The *nad5* minichromosome was significantly higher in transcription level than *trnW-trnS*_2_ minichromosome (*p* < 0.05) (Figure 2, Table S9).

A similar pattern was also seen when only the data sets of *P. humanus capitis* were analysed. The *trnL*_2_*-rrnL* minichromosome had a significantly higher transcription level than seven other minichromosomes: *trnG-nad4L-trnV* (*p* < 0.05), *trnL*_1_*-rrnS-trnC* (*p* < 0.01), *trnM* (*p* < 0.001), *trnP-nad2-trnI* (*p* = 0.015), *trnS*_1_*-trnN-trnE* (*p* < 0.01), *trnT-trnD-trnH* (*p* < 0.0001), and *trnW-trnS*_2_ (*p* < 0.00001) (Figure S1, Table S10). The *cox1* minichromosome had a significantly higher transcription level than five other minichromosomes: *trnL*_1_*-rrnS-trnC* (*p* < 0.05), *trnM* (*p* < 0.001), *trnS*_1_*-trnN-trnE* (*p* < 0.05), *trmT-trnD-trnH* (*p* < 0.001), and *trnW-trnS*_2_ (*p* < 0.00005) (Figure S1, Table S10). The *trnY-cox2* minichromosome had a significantly higher transcription level than 3 other minichromosomes: *trnM* (*p* < 0.005), *trnT-trnD-trnH* (*p* < 0.005), and *trnW-trnS*_2_ (*p* < 0.0005) (Figure S1, Table S10). The *atp8-atp6* minichromosome, *cox3-trnA* minichromosome and *trnK-nad4* minichromosome had significantly higher transcription levels than two other minichromosomes: *trnT-trnD-trnH* (*p* < 0.05), and *trnW-trnS*_2_ (*p* < 0.05) (Figure S1, Table S10). The *cob* minichromosome had a significantly higher transcription level than the *trnW-trnS*_2_ minichromosome (*p* < 0.05) (Figure S1, Table S10).

### 3.4. No Transcription Initiation Sites Can Be Located at the Conserved AT-Rich Motifs, but Many Transcription Events Appear to Terminate near the Conserved GC-Rich Motifs

**Figure 3:**
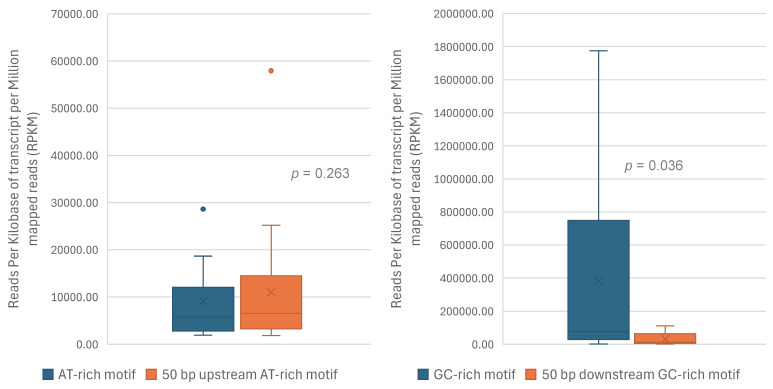
Comparison of transcription level between AT-rich motif and its upstream 50 bp sequence (**left**), and between GC-rich motif and its downstream 50 bp sequence (**right**). Eleven mitochondrial minichromosomes of the human body louse, *P. humanus corporis*, were available for comparison using the Wilcoxon test: *atp8-atp6*, *cob*, *cox1*, *trnY-cox2*, *cox3-trnA*, *trnP-nad2-trnI*, *trnR-nad3*, *trnK-nad4*, *nad5*, *trnL*_1_*-rrnS-trnC* and *trnL*_2_*-rrnL* (Table S4). The *p*-value is displayed for each pair of comparisons.

Both *P. humanus capitis* and *P. humanus corporis* have an AT-rich motif and a GC-rich motif in the non-coding region of each mt minichromosome; the AT-rich motif is upstream of the coding region, whereas the GC-rich motif is downstream of the coding region [14,15]. These motifs are highly conserved among all the minichromosomes of each subspecies and between the two subspecies, and thus, may potentially be linked to transcription and replication of mt minichromosomes [15]. We investigated the possibility of the AT-rich motifs being the transcription initiation sites and the GC-rich motifs being the transcription termination sites. The Wilcoxon tests showed that the transcription level of each AT-rich motif was not significantly different from that of the 50 bp sequence immediately upstream from the AT-rich motif (*p* = 0.263, Figure 3, Table S11), providing no support for the possibility that these AT-rich motifs were transcription initiation sites. In contrast, the transcription level of the GC-rich motif was significantly higher than that of the 50 bp sequence immediately downstream from the GC-rich motif (*p* = 0.036, Figure 3, Table S11), which supported the possibility that these GC-rich motifs might serve as transcription termination sites. Not all transcription events, however, terminated at these GC-rich motifs; some transcription events continued after these motifs, thus leading to the transcription of the entire mt minichromosomes described above in Section 3.2.

### 3.5. Transcription Level Varies Among Mitochondrial Protein-Coding and rRNA Genes

**Figure 4:**
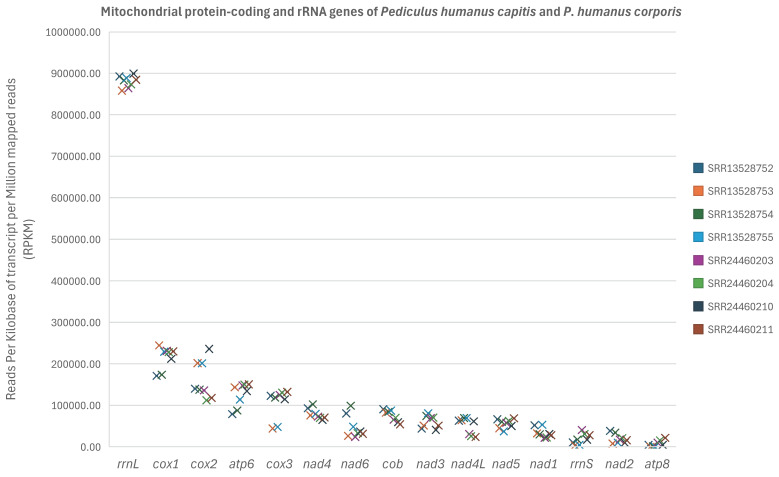
Comparison of transcription level among mitochondrial protein-coding and rRNA genes of the human head louse, *Pediculus humanus capitis*, and the human body louse, *P. humanus corporis*, using the Friedman test. The *p* values are provided in Table S12 for each pair of mitochondrial genes. Details of the eight RNA-seq data sets are in Table S1.

Comparison among mt protein-coding and rRNA genes of *P. humanus capitis* and *P. humanus corporis* using the Friedman test showed that *rrnL* was significantly higher in transcription level than *rrnS* (*p* < 0.00005), *atp8* (*p* < 0.0000001), *nad1* (*p* < 0.001), *nad2* (*p* < 0.00001), *nad4L* (*p* < 0.01), *nad5* (*p* < 0.05) and *nad6* (*p* < 0.01) (Figure 4, Table S12). *cox1* was significantly higher than *rrnS* (*p* < 0.0005), *atp8* (*p* < 0.000005), *nad1* (*p* < 0.005), and *nad2* (*p* < 0.00005). *cox2* was significantly higher than *rrnS* (*p* < 0.005), *atp8* (*p* < 0.00005), *nad1* (*p* < 0.05), and *nad2* (*p* < 0.001). *atp6* was significantly higher than *rrnS* (*p* < 0.05), *atp8* (*p* < 0.0005), and *nad2* (*p* < 0.01). *cob*, *cox3* and *nad4* were significantly higher than *atp8* (*p* < 0.05) (Figure 4, Table S12).

A consistent pattern was also seen when only the data sets of *P. humanus capitis* were analysed using the Friedman test. *rrnL* was significantly higher in transcription level than *rrnS* (*p* < 0.0005), *atp8* (*p* < 0.00001), *nad1* (*p* < 0.005), and *nad2* (*p* < 0.0005) (Figure S2, Table S13). *cox1* was significantly higher in transcription level than four genes: *rrnS* (*p* < 0.005), *atp8* (*p* = 0.00005), *nad1* (*p* < 0.05) and *nad2* (*p* < 0.005). *cox2* was significantly higher in transcription level than *rrnS* (*p* < 0.05), *atp8* (*p* < 0.001), and *nad2* (*p* = 0.019); *atp6* was significantly higher than *atp8* (*p* < 0.01) (Figure S2, Table S13).

### 3.6. Transcription Initiation Site for atp6 Gene Is Located at the 3′ End of the Upstream atp8 Gene

**Figure 5:**
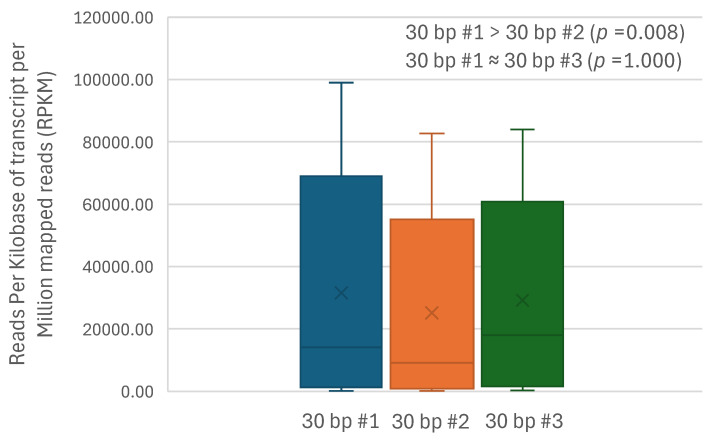
Comparison of transcription level: (1) Between 30 bp sequence #1 (at the 3′ end of *atp8* immediately before *atp6*) and 30 bp sequence #2 (upstream of 30 bp #1), and (2) between 30 bp #1 and 30 bp sequence #3 (downstream of 30 bp #1 at the 5′ end of *atp6*) of the *atp8*-*atp6* minichromosomes of the human head louse, *Pediculus humanus capitis*, and the human body louse, *P. humanus corporis*, using the Friedman test (Table S14).

The observation above that *atp6* is significantly higher than *atp8* in transcription level is noteworthy because these two genes are together as a cluster, *atp8*-*atp6*, in the same mt minichromosome [14,15]. This observation indicates that *atp6* and *atp8* are transcribed separately; *atp6* most likely has its own transcription initiation site because it is downstream of *atp8*. We compared the transcription levels using the Friedman test: (1) between the 30 bp sequence at the 3′ end of *atp8* immediately before *atp6* (named 30 bp #1) and 30 bp sequence #2, which is upstream of 30 bp #1; and (2) between 30 bp #1 and 30 bp sequence #3, which is downstream of 30 bp #1 at the 5′ end of *atp6* (Table S3). From the results, 30 bp #1 was significantly higher in transcription level than its upstream 30 bp #2 (*p* = 0.008) but had no significant difference from its downstream 30 bp #3 (*p* = 1.000, Figure 5, Table S14). These results support 30 bp #1 at the 3′ end of *atp8* as a potential initiation site for the transcription of the *atp6* gene.

### 3.7. No Significant Difference in Transcription Level Among Most Mitochondrial tRNA Genes

**Figure 6:**
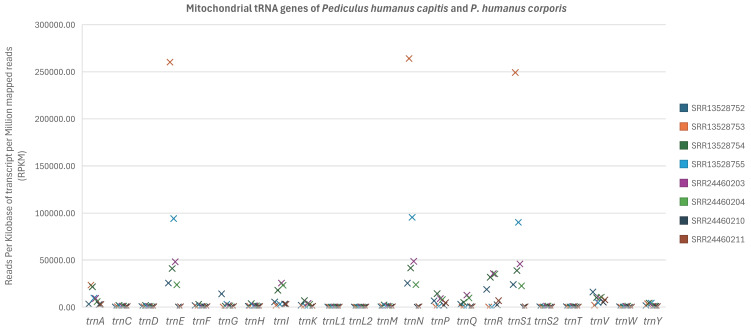
Comparison of transcription level among mitochondrial tRNA genes of the human head louse, *Pediculus humanus capitis*, and the human body louse, *P. humanus corporis*, using the Friedman test. The *p*-values are provided in Table S15 for each pair of mitochondrial genes. Details of the eight RNA-seq data sets are in Table S1.

No significant difference in transcription level was revealed among most of the mt tRNA genes of *P. humanus capitis* and *P. humanus corporis* with the Friedman test. The only exception was that *trnL*_1_ and *trnL*_2_ were significantly lower in transcription level than *trnR* (*p* < 0.05, Figure 6, Table S15). A similar pattern was also seen when only the data sets of *P. humanus capitis* were analysed. Both *trnL*_1_ and *trnL*_2_ were significantly lower in transcription level than *trnN* (*p* < 0.05), and *trnL*_1_ was significantly lower in transcription level than *trnE* (*p* < 0.05, Figure S3, Table S16). *trnN* and *trnE* are located on the same mt minichromosome as a cluster in both *Pediculus* subspecies [14,15].

## 4. Discussion

Transcription of the single-chromosome mt genomes typical of eukaryotes was best studied in humans and was reviewed in detail in Taanman [1]. The human mt genome has three transcription initiation sites and a single transcription termination site. Two transcription initiation sites, IT_H1_ and IT_H2_, are for the heavy strand (H-strand), which contains 28 of the total 37 mt genes; one initiation site, IT_L_, is for the light strand (L-strand), which contains the other nine mt genes. IT_H1_ and IT_L_ are the major initiation sites, both located in the non-coding region called D-loop, 150 bp apart from one another. Both IT_H1_ and IT_L_ are surrounded by a 15 bp A-rich promoter element (5′-CANACC(G)CC(A)AAAGAYA-3′), critical for transcription. IT_H2_ is an additional transcription initiation site for the H-strand, located 77 bp downstream of IT_H1_, at the 3′ end of the *trnF* gene and immediately before the 5′ end of the *rrnS* gene. The transcription termination site in the human mt genome is 28 bp long, starting from the last nucleotide of the *rrnL* gene and ending at the 27th nucleotide of the *trnL*_2_ gene [29]. A 13 bp GC-rich motif (5′-TGGCAGAGCCCGG-3′) in this site is critical for transcription termination [29]. A protein called mt transcription terminator (mtTERM) can bind to this site on either the H-strand or L-strand and terminate transcription [29]. Most transcription initiated from IT_H1_ terminates at the mtTERM binding site, producing a transcript for tRNA^Phe^, tRNA^Val^ and two rRNAs; most transcription initiated from IT_H2_, however, does not stop at the mtTERM binding site and produces a transcript for all mRNAs, rRNAs and tRNAs coded by the H-strand. Where exactly the transcription initiated from IT_H2_ terminates is not clear; potentially, the entire H-strand can be transcribed. Transcription initiation is much more frequent from IT_H1_ than from IT_H2_, resulting in a higher abundance (50–100 fold) of transcripts for tRNA^Phe^, tRNA^Val^ and two rRNAs than for other tRNAs and mRNAs coded by the H-strand [25,29]. Most transcription initiated from IT_L_ also terminates at the mtTERM binding site, producing a transcript for eight tRNAs and an mRNA for the NAD6 protein coded by the L-strand [1]. The entire L-strand can be transcribed, including the section downstream of mtTERM binding site till IT_L_; this section does not contain any genes. mtTERM termination is more efficient (two- to three-fold) for the L-strand transcription (50–70% terminated) than for the H-strand transcription (20–30% terminated) [29]; thus, the L-strand section from the mtTERM binding site to IT_L_ is likely the least transcribed section in the human mt genome.

Our results revealed several similar features in transcription between the extensively fragmented mt genomes of the human lice and the human mt genome. First, the entire mt minichromosomes of the human lice, including both coding and non-coding regions, are transcribed. This matches the observation described above that both the H-strand and L-strand of the human mt genome can be transcribed entirely. Second, transcription level varies significantly between coding and non-coding regions, among different mt minichromosomes, and among different mt genes of the human lice. This is consistent with the observation in the human mt genome that: (1) transcripts for tRNA^Phe^, tRNA^Val^ and two rRNAs (initiated from IT_H1_) are 50–100 fold more abundant than those for other tRNAs and mRNAs coded by the H-strand (initiated from IT_H2_); and (2) the L-strand section from mtTERM binding site to IT_L_ is transcribed at a much lower level than other sections of the mt genome due to the efficient mtTERM termination of the L-strand transcription [25,29]. Third, transcription initiation sites can be located at the 3′ end of the upstream gene. In the human lice, the transcription initiation site for *atp6* is likely located at the 3′ end of the upstream *atp8* gene. In the human mt genome, IT_H2_ is at the 3′ end of the *trnF* gene. Fourth, many transcription events terminate at the conserved GC-rich motif downstream of the coding region in the human lice. This motif has 58% G and C and contains multiple strings of G and C [15]. In the human mt genome, the transcription termination site contains a 13 bp motif with 77% G and C, which is critical for transcription termination [29]. Given that the GC-richness is conserved between humans and human lice, it would be reasonable to postulate that GC-rich motifs are likely a critical component for the transcription termination sites of other animals.

On the other hand, our results showed several distinct features in human lice relative to humans in mt genome transcription, apparently due to their drastic difference in mt genome organisation. First, each of the 20 mt minichromosomes of human lice likely has its own transcription termination site at the conserved GC-rich motif, in contrast to the single transcription termination site for the whole mt genome in humans. Although we could not identify transcription initiation sites for all genes in human lice in the current study, we assume that each mt minichromosome of human lice should have its own transcription initiation site(s) to work with its termination site, which is the case for the *atp6* gene. Second, there is much more variation in transcription level among different genes in human lice than in humans. In the human mt genome, all the genes on the L-strand are transcribed together, and thus, would be in equal abundance; so are all the genes on the H-strand except for the genes for tRNA^Phe^, tRNA^Val^ and two rRNAs, which are transcribed separately in much higher abundance than other genes. In human lice, each mt minichromosome is apparently transcribed separately, which explains the significant variation in transcription level among different minichromosomes and among different genes we observed in the current study. Third, there is a significant difference in transcription level between the two rRNA genes in human lice: *rrnL* is significantly higher than *rrnS* (*p* < 0.0005) (Figure 4). This can be attributed to the fact that *rrnL* and *rrnS* are on different minichromosomes in human lice and, thus, are transcribed separately. In the human mt genome, the two rRNA genes are transcribed together in equal abundance, which makes sense for the formation of mt ribosomes. A higher transcription level of rRNA genes than other genes has also been observed in other vertebrate mitochondria [4,25,26]. This is also the case in the nuclear genomes of eukaryotes, such as humans and yeasts: the large subunit (60S) and the small subunit (40S) are equally expressed, as both the large and small subunits are used together to translate mRNAs into proteins [30]. The significant difference in transcription level between the two mt rRNA genes in human lice indicates that the transcription level among different mt minichromosomes is not regulated in a coordinated manner.

## 5. Conclusions

The current study represents the first attempt to understand the transcription of fragmented mt genomes with RNA-seq data, for which the human louse can serve as a valuable model. The ever-increasing RNA-seq data provide a rich resource for such efforts. The methods we used in the current study can be a stepping stone for further studies. Our analyses revealed similar features in transcription shared between human lice and humans, and the unique features in human lice due to their extensively fragmented mt genomes. A major limitation of the current study was the relatively small amount, but rather heterogeneous, of RNA-seq data sets available now for human lice. This can impact the power of statistical analysis and make it difficult to determine small differences in transcription level between genes, between minichromosomes, between coding and non-coding regions, and between the two subspecies. Further analysis using a larger sample size is certainly necessary as more data become available. Differences in the sequencing strategy used in different RNA-seq data sets, e.g., whether mRNAs are enriched or not, can also impact comparisons and results, as insect mitochondrial transcripts are polyadenylated [31]. Studies using data sets generated with the same sequencing strategy would provide clearer and more accurate comparisons. Strand-specific RNA-seq data are not currently available for human lice. The general RNA-seq data sets currently available for human lice cannot differentiate the transcription level between the sense strand and the non-sense strand. Thus, the results and conclusions of the current study should be taken in the context of general RNA-seq data sets only (i.e., not strand-specific). The current study was limited to human head and body lice only and, therefore, cannot be generalised to other parasitic lice or other animals with fragmented mt genomes. Mitochondrial genome fragmentation occurred more than 30 times in parasitic lice [32], resulting in a substantial variation in mt genome organisation, number of minichromosomes, and mt gene arrangement among parasitic lice [16,20,21,22]. Each of these fragmentation events is likely accompanied by unique changes in mt genome transcription. Further studies on other parasitic lice, other animals or eukaryotes with fragmented mt or chloroplast genomes are necessary to fully elucidate how organelle genome fragmentation may impact the transcription of these genomes. Last but not least, findings made from RNA-seq data analyses need to be validated by experimental approaches.

## Figures and Tables

**Table 1:** Adjusted significance values from Friedman’s two-way analysis of variance by ranks of the mitochondrial gene expression level among the eight RNA-seq data sets of human lice retrieved from the NCBI SRA database.

	SRR13528753 (Female, 0-day-old ovary, *P. humanus capitis* (head louse))	SRR13528754 (Female, 5-day-old accessory gland, *P. humanus capitis* (head louse))	SRR13528755 (Female, 0-day-old accessory gland, *P. humanus capitis* (head louse))	SRR24460203 (Male, tissue not specified, *P. humanus capitis* (head louse))	SRR24460204 (Male, tissue not specified, *P. humanus capitis* (head louse))	SRR24460210 (Male, tissue not specified, *P. humanus corporis* (body louse))	SRR24460211 (Male, tissue not specified, *P. humanus corporis* (body louse))
SRR13528752 (Female, 5-day-old ovary, *Pediculus humanus capitis* (head louse))	0.024 *	0.903	1.000	1.000	1.000	0.037 *	1.000
SRR13528753 (Female, 0-day-old ovary, *P. humanus capitis* (head louse))		0.0000012 *	0.817	0.00017 *	0.00017 *	1.000	1.000
SRR13528754 (Female, 5-day-old accessory gland, *P. humanus capitis* (head louse))			<0.028 *	1.000	1.000	0.0000024 *	<0.010 *
SRR13528755 (Female, 0-day-old accessory gland, *P. humanus capitis* (head louse))				0.514	0.541	1.000	1.000
SRR24460203 (Male, tissue not specified, *P. humanus capitis* (head louse))					1.000	0.00030 *	<0.248
SRR24460204 (Male, tissue not specified, *P. humanus capitis* (head louse))						<0.00030 *	<0.248
SRR24460210 (Male, tissue not specified, *P. humanus corporis* (body louse))							1.000

Note: * indicates *p* < 0.05.

## Data Availability

All the RNA-seq data analysed in the article are available in the NCBI SRA database as described in Section 2.

## Supplementary Materials

The following supporting information can be downloaded at: https://www.mdpi.com/article/10.3390/biology15040296/s1, Figure S1: Comparison of transcription level among mitochondrial minichromosomes of the human head louse, *Pediculus humanus capitis*, using the Friedman test. The *p*-values are provided in Table S10 for each pair of mitochondrial minichromosomes. Figure S2: Comparison of transcription level among mitochondrial protein-coding and rRNA genes of the human head louse, *Pediculus humanus capitis*, using the Friedman test. The *p*-values are provided in Table S13 for each pair of mitochondrial genes. Figure S3: Comparison of transcription level among mitochondrial tRNA genes of the human head louse, *Pediculus humanus capitis*, using the Friedman test. The *p*-values are provided in Table S16 for each pair of mitochondrial tRNA genes. Table S1: RNA-seq data sets of the human lice retrieved from NCBI SRA database and analysed in the current study. Table S2: The mitochondrial minichromosomes, genes and long non-coding regions of the human lice investigated in the current study. Table S3: RPKM and TRM for each mitochondrial gene, minichromosome, and motif of the human head louse, *Pediculus humanus capitis*, and the human body louse, *Pediculus humanus corporis*. Table S4: RPKM and TPM of the AC-rich motif and the GC-rich motif in the mitochondrial minichromosomes of the human body louse, *Pediculus humanus corporis*. Table S5: Mean read coverage of the coding and non-coding regions of each mitochondrial minichromosome of the human head louse, *Pediculus humanus capitis*, and the human body louse, *Pediculus humanus corporis*. Table S6: Normality tests of each RNA-seq data set of the human head louse, *Pediculus humanus capitis*, and the human body louse, *Pediculus humanus corporis*. Table S7: Comparison between the coding and non-coding region of each mitochondrial minichromosome of the human head louse, *Pediculus humanus capitis*, using the Wilcoxon signed-ranks test. Table S8: Comparison between the coding and non-coding region of each mitochondrial minichromosome of the human body louse, *Pediculus humanus corporis*, using the Wilcoxon signed-ranks test. Table S9: Pair-wise comparison of transcription level between mitochondrial minichromosomes of the human head louse, *Pediculus humanus capitis*, and the human body louse, *Pediculus humanus capitis*, using the Friedman test. Table S10: Pair-wise comparison of transcription level between mitochondrial minichromosomes of the human head louse, *Pediculus humanus capitis*, using the Friedman test. Table S11: Pair-wise comparison of transcriptional level between AT-rich motif and its upstream 50 bp sequence, and between GC-rich motif and its downstream 50 bp sequence of the human body louse, *Pediculus humanus corporis*, using the Wilcoxon test. Table S12: Pair-wise comparison of transcription level between mitochondrial protein-coding and rRNA genes of the human head louse, *Pediculus humanus capitis*, and the human body louse, *Pediculus humanus capitis*, using the Friedman test. Table S13: Pair-wise comparison of transcription level between mitochondrial protein-coding and rRNA genes of the human head louse, *Pediculus humanus capitis*, using the Friedman test. Table S14: Comparison of the transcription level among 30 bp sequence #1 (immediately before *atp6* gene), 30 bp sequence #2 (upstream of #1) and 30 bp sequence #3 (downstream of #1) in the human head louse, *Pediculus humanus capitis*, and the human body louse, *Pediculus humanus capitis*, using the Friedman test. Table S15: Pair-wise comparison of transcription level between mitochondrial tRNA genes of the human head louse, *Pediculus humanus capitis*, and the human body louse, *Pediculus humanus capitis*, using the Friedman test. Table S16: Pair-wise comparison of transcription level between mitochondrial tRNA genes of the human head louse, *Pediculus humanus capitis*, using the Friedman test.

## Abbreviations

The following abbreviations are used in this manuscript:

mt	mitochondrial
RPKM	Reads Per Kilobase of transcript per Million mapped reads
TPM	Transcripts Per Million
SRA	Sequence Read Archive
mtTERM	mt transcription terminator

## References

[1] Taanman J.W. (1999). The Mitochondrial Genome: Structure, Transcription, Translation and Replication. Biochim. Biophys. Acta.

[2] Garcia L.E., Sanchez-Puerta M.V. (2021). Transcriptional Landscape and Splicing Efficiency in Arabidopsis Mitochondria. Cells.

[3] Basu U., Bostwick A.M., Das K., Dittenhafer-Reed K.E., Patel S.S. (2020). Structure, Mechanism, and Regulation of Mitochondrial DNA Transcription Initiation. J. Biologol. Chem..

[4] Boore J.L. (1999). Animal Mitochondrial Genomes. Nucleic Acids Res..

[5] Lavrov D.V., Pett W., Voigt O., Wörheide G., Forget L., Lang B.F., Kayal E. (2013). Mitochondrial DNA of *Clathrina clathrus* (Calcarea, Calcinea): Six Linear Chromosomes, Fragmented rRNAs, tRNA Editing, and a Novel Genetic Code. Mol. Biol. Evol..

[6] Lavrov D.V., Adamski M., Chevaldonné P., Adamska M. (2016). Extensive Mitochondrial mRNA Editing and Unusual Mitochondrial Genome Organization in Calcaronean Sponges. Curr. Biol..

[7] Voigt O., Erpenbeck D., Wörheide G. (2008). A Fragmented Metazoan Organellar Genome: The Two Mitochondrial Chromosomes of *Hydra magnipapillata*. BMC Genom..

[8] Smith D.R., Kayal E., Yanagihara A.A., Collins A.G., Pirro S., Keeling P.J. (2012). First Complete Mitochondrial Genome Sequence from a Box Jellyfish Reveals a Highly Fragmented Linear Architecture and Insights into Telomere Evolution. Genome Biol. Evol..

[9] Suga K., Mark Welch D.B., Tanaka Y., Sakakura Y., Hagiwara A. (2008). Two Circular Chromosomes of Unequal Copy Number Make up the Mitochondrial Genome of the Rotifer *Brachionus plicatilis*. Mol. Biol. Evol..

[10] Watanabe K.I., Bessho Y., Kawasaki M., Hori H. (1999). Mitochondrial Genes Are Found on Minicircle DNA Molecules in the Mesozoan Animal *Dicyema*. J. Mol. Biol..

[11] Armstrong M.R., Blok V.C., Phillips M.S. (2000). A Multipartite Mitochondrial Genome in the Potato Cyst Nematode *Globodera pallida*. Genetics.

[12] Wei D.-D., Shao R., Yuan M.-L., Dou W., Barker S.C., Wang J.-J. (2012). The Multipartite Mitochondrial Genome of *Liposcelis bostrychophila*: Insights into the Evolution of Mitochondrial Genomes in Bilateral Animals. PLoS ONE.

[13] Perlman S.J., Hodson C.N., Hamilton P.T., Opit G.P., Gowen B.E. (2015). Maternal Transmission, Sex Ratio Distortion, and Mitochondria. Proc. Natl. Acad. Sci. USA.

[14] Shao R., Kirkness E.F., Barker S.C. (2009). The Single Mitochondrial Chromosome Typical of Animals Has Evolved into 18 Minichromosomes in the Human Body Louse, *Pediculus humanus*. Genome Res..

[15] Shao R., Zhu X.-Q., Barker S.C., Herd K. (2012). Evolution of Extensively Fragmented Mitochondrial Genomes in the Lice of Humans. Genome Biol. Evol..

[16] Cameron S.L., Yoshizawa K., Mizukoshi A., Whiting M.F., Johnson K.P. (2011). Mitochondrial Genome Deletions and Minicircles Are Common in Lice (Insecta: Phthiraptera). BMC Genom..

[17] Jiang H., Barker S.C., Shao R. (2013). Substantial Variation in the Extent of Mitochondrial Genome Fragmentation among Blood-Sucking Lice of Mammals. Genome Biol. Evol..

[18] Herd K.E., Barker S.C., Shao R. (2015). The Mitochondrial Genome of the Chimpanzee Louse, *Pediculus schaeffi*: Insights into the Process of Mitochondrial Genome Fragmentation in the Blood-Sucking Lice of Great Apes. BMC Genom..

[19] Song F., Li H., Liu G.-H., Wang W., James P., Colwell D.D., Tran A., Gong S., Cai W., Shao R. (2019). Mitochondrial Genome Fragmentation Unites the Parasitic Lice of Eutherian Mammals. Syst. Biol..

[20] Sweet A.D., Johnson K.P., Cameron S.L. (2022). Independent Evolution of Highly Variable, Fragmented Mitogenomes of Parasitic Lice. Commun. Biol..

[21] Dong Y., Jelocnik M., Gillett A., Valenza L., Conroy G., Potvin D., Shao R. (2023). Mitochondrial Genome Fragmentation Occurred Multiple Times Independently in Bird Lice of the Families Menoponidae and Laemobothriidae. Animals.

[22] Najer T., Doña J., Buček A., Sweet A.D., Sychra O., Johnson K.P. (2024). Mitochondrial Genome Fragmentation Is Correlated with Increased Rates of Molecular Evolution. PLoS Genet..

[23] Morozov Y.I., Parshin A.V., Agaronyan K., Cheung A.C.M., Anikin M., Cramer P., Temiakov D. (2015). A Model for Transcription Initiation in Human Mitochondria. Nucleic Acids Res..

[24] Tan B.G., Gustafsson C.M., Falkenberg M. (2024). Mechanisms and Regulation of Human Mitochondrial Transcription. Nat. Rev. Mol. Cell Biol..

[25] Gelfand R., Attardi G. (1981). Synthesis and Turnover of Mitochondrial Ribonucleic Acid in HeLa Cells: The Mature Ribosomal and Messenger Ribonucleic Acid Species Are Metabolically Unstable. Mol. Cell Biol..

[26] Held J.P., Patel M.R. (2020). Functional Conservation of Mitochondrial RNA Levels despite Divergent mtDNA Organization. BMC Res. Notes.

[27] Wang Z., Gerstein M., Snyder M. (2009). RNA-Seq: A Revolutionary Tool for Transcriptomics. Nat. Rev. Genet..

[28] Head S.R., Komori H.K., LaMere S.A., Whisenant T., Van Nieuwerburgh F., Salomon D.R., Ordoukhanian P. (2014). Library Construction for Next-Generation Sequencing: Overviews and Challenges. Biotechniques.

[29] Shang J., Clayton D.A. (1994). Human Mitochondrial Transcription Termination Exhibits RNA Polymerase Independence and Biased Bipolarity in Vitro. J. Biol. Chem..

[30] Gregory B., Rahman N., Bommakanti A., Shamsuzzaman M., Thapa M., Lescure A., Zengel J.M., Lindahl L. (2019). The Small and Large Ribosomal Subunits Depend on Each Other for Stability and Accumulation. Life Sci. Alliance.

[31] Bratic A., Clemente P., Calvo-Garrido J., Maffezzini C., Felser A., Wibom R., Wedell A., Freyer C., Wredenberg A. (2016). Mitochondrial Polyadenylation Is a One-Step Process Required for mRNA Integrity and tRNA Maturation. PLoS Genet..

[32] Song N., Shao R. (2025). Loss of Mitochondrial Single Stranded DNA-Binding Protein (mtSSB) Gene Is Associated with Mitochondrial Genome Fragmentation in Psocodea (Bark Lice, Book Lice, and Parasitic Lice). BMC Biol..

