# Supplementary material for: Transcription of the Extensively Fragmented Mitochondrial Genomes of Human Lice
Source: Biology (Basel). 2026 Feb 8;15(4):296. doi: 10.3390/biology15040296 (PMC12938707; doi:10.3390/biology15040296)
Supplement: Supplementary file 1 [file biology-15-00296-s001.zip › Supplementary Figure S3.pdf]

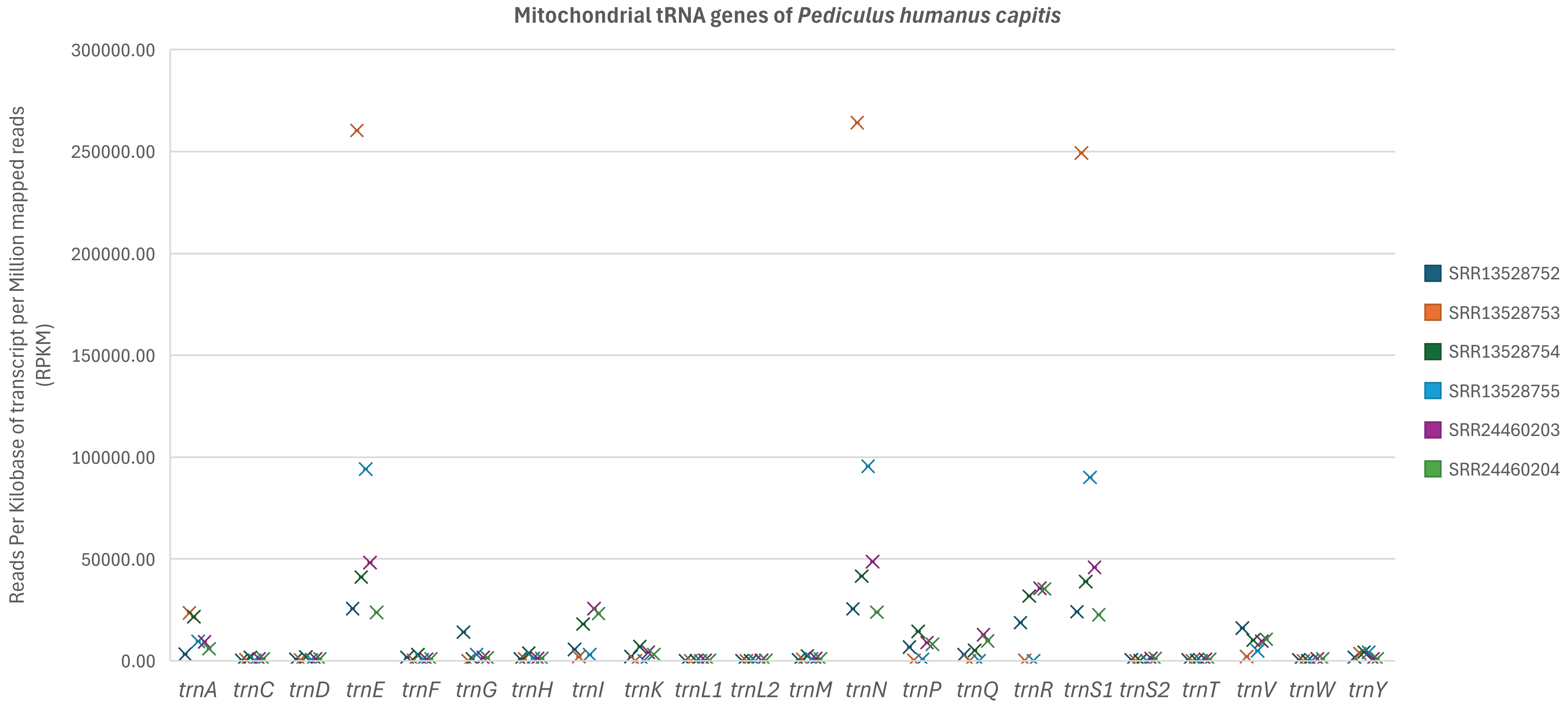


**Figure S3**: Comparison of transcription level among mitochondrial tRNA genes of the human head louse, *Pediculus humanus capitis*, using the Friedman test. The P values are provided in Table S16 for each pair of mitochondrial tRNA genes.
