# Supplementary material for: Transcription of the Extensively Fragmented Mitochondrial Genomes of Human Lice
Source: Biology (Basel). 2026 Feb 8;15(4):296. doi: 10.3390/biology15040296 (PMC12938707; doi:10.3390/biology15040296)
Supplement: Supplementary file 1 [file biology-15-00296-s001.zip › Supplementary Table S1.pdf]

Table S1: RNA-seq data sets of the human lice retrieved from NCBI SRA database and analysed in the current study.

Data set number	Number of reads	Library information	Date published	Subspecies	Sex	Tissue	Study	Submitted by	NCBI link
SRR13528752	3,416,294	**Design:** Paired end sequencing **Instrument:** Illumina NovaSeq 6000 **Strategy:** RNA-Seq **Source:** Transcriptomic **Selection:** Oligo-dT	26 Jan 2021	*Pediculus humanus capitis* (head louse)	Female	5-day-old ovary	Tissue-specific gene expression in reproductive systems of female head louse	Seoul National University	https://www.ncbi.nlm.nih.gov/sra/SRX9937075[accn]
SRR13528753	2,513,869	**Design:** Paired end sequencing **Instrument:** Illumina NovaSeq 6000 **Strategy:** RNA-Seq **Source:** Transcriptomic **Selection:** Oligo-dT	26 Jan 2021	*Pediculus human's capitis* (head louse)	Female	0-day-old ovary	Tissue-specific gene expression in reproductive systems of female head louse	Seoul National University	https://www.ncbi.nlm.nih.gov/sra/SRX9937074[accn]
SRR13528754	493,990	**Design:** Paired end sequencing **Instrument:** Illumina NovaSeq 6000 **Strategy:** RNA-Seq **Source:** Transcriptomic **Selection:** Oligo-dT	26 Jan 2021	*Pediculus humanus capitis* (head louse)	Female	5-day-old accessory gland	Tissue-specific gene expression in reproductive systems of female head louse	Seoul National University	https://www.ncbi.nlm.nih.gov/sra/SRX9937073[accn]
SRR13528755	2,548,225	**Design:** Paired end sequencing **Instrument:** Illumina NovaSeq 6000 **Strategy:** RNA-Seq **Source:** Transcriptomic **Selection:** Oligo-dT	26 Jan 2021	*Pediculus humanus capitis* (head louse)	Female	0-day-old accessory gland	Tissue-specific gene expression in reproductive systems of female head louse	Seoul National University	https://www.ncbi.nlm.nih.gov/sra/SRX9937072[accn]
SRR24460203	11,107,132	**Design:** TruSeq stranded 50bp **Instrument:** Illumina HiSeq 4000 **Strategy:** RNA-Seq **Source:** Transcriptomic	7 May 2023	*Pediculus humanus capitis* (head louse)	Male	Not specified	Lack of paternal silencing and ecotype specific expression in head and body louse hybrids	University of Leicester	https://www.ncbi.nlm.nih.gov/sra/SRX20247150[accn]

		**Selection:** Random							
SRR24460204	7,962,181	**Design:** TruSeq stranded 50bp<br>**Instrument:** Illumina HiSeq 4000<br>**Strategy:** RNA-Seq<br>**Source:** Transcriptomic<br>**Selection:** Random	7 May 2023	*Pediculus humanus capitis* (head louse)	Male	Not specified	Lack of paternal silencing and ecotype specific expression in head and body louse hybrids	University of Leicester	https://www.ncbi.nlm.nih.gov/sra/SRX20247149[accn]
SRR24460210	11,458,109	**Design:** TruSeq stranded 50bp<br>**Instrument:** Illumina HiSeq 4000<br>**Strategy:** RNA-Seq<br>**Source:** Transcriptomic<br>**Selection:** Random	7 May 2023	*Pediculus humanus corporis* (body louse)	Male	Not specified	Lack of paternal silencing and ecotype specific expression in head and body louse hybrids	University of Leicester	https://www.ncbi.nlm.nih.gov/sra/SRX20247143[accn]
SRR24460211	6,459,189	**Design:** TruSeq stranded 50bp<br>**Instrument:** Illumina HiSeq 4000<br>**Strategy:** RNA-Seq<br>**Source:** Transcriptomic<br>**Selection:** Random	7 May 2023	*Pediculus humanus corporis* (body louse)	Male	Not specified	Lack of paternal silencing and ecotype specific expression in head and body louse hybrids	University of Leicester	https://www.ncbi.nlm.nih.gov/sra/SRX20247142[accn]
