# Supplementary material for: Transcription of the Extensively Fragmented Mitochondrial Genomes of Human Lice
Source: Biology (Basel). 2026 Feb 8;15(4):296. doi: 10.3390/biology15040296 (PMC12938707; doi:10.3390/biology15040296)
Supplement: Supplementary file 1 [file biology-15-00296-s001.zip › Supplementary Table S11.pdf]

Table S11: Pair-wise comparison of transcriptional level (RPKM) between AT-rich motif and its upstream 50-bp sequence, and between GC-rich motif and its downstream 50-bp sequence of the human body louse, *Pediculus humanus corporis*, using the Wilcoxon test.

Ranks

		N	Mean Rank	Sum of Ranks
50 bp downstream GC-rich motif - GC-rich motif	Negative Ranks	12[a]	15.92	191.00
	Positive Ranks	10[b]	6.20	62.00
	Ties	0[c]		
	Total	22		
50 bp upstream AT-rich motif - AT-rich motif	Negative Ranks	8[d]	11.50	92.00
	Positive Ranks	14[e]	11.50	161.00
	Ties	0[f]		
	Total	22		

a. 50 bp downstream GC-rich motif < GC-rich motif

b. 50 bp downstream GC-rich motif > GC-rich motif

c. 50 bp downstream GC-rich motif = GC-rich motif

d. 50 bp upstream AT-rich motif < AT-rich motif

e. 50 bp upstream AT-rich motif > AT-rich motif

f. 50 bp upstream AT-rich motif = AT-rich motif

Test Statistics[a]

	50 bp downstream GC-rich motif - GC-rich motif	50 bp upstream AT-rich motif - AT-rich motif
Z	-2.094[b]	-1.120[c]
Asymp. Sig. (2-tailed)	0.036	0.263

a. Wilcoxon Signed Ranks Test

b. Based on positive ranks.

c. Based on negative ranks.
