# Supplementary material for: Transcription of the Extensively Fragmented Mitochondrial Genomes of Human Lice
Source: Biology (Basel). 2026 Feb 8;15(4):296. doi: 10.3390/biology15040296 (PMC12938707; doi:10.3390/biology15040296)
Supplement: Supplementary file 1 [file biology-15-00296-s001.zip › Supplementary Table S2.pdf]

Table S2: The mitochondrial minichromosomes, genes and long non-coding regions of the human lice investigated in the current study.

Minichromosome	Gene	Gene product	Gene position	Length (bp)
atp8-atp6	*atp8*	ATP synthase subunit 8	Head[a]: 77-268<br>Body[b]: 156-347	Head: 192<br>Body: 192
	atp6	ATP synthase subunit 6	Head: 272-922<br>Body: 351-1001	Head: 651<br>Body: 651
	$LNR_{atp8\text{-}atp6}$	Long non-coding RNA		Body: 2107
cob	*cob*	Cytochrome b	Head: 117-1127<br>Body: 196-1206	Head: 1011<br>Body: 1011
	LNR_{cob}	Long non-coding RNA		Body: 2313
cox1	*cox1*	Cytochrome c oxidase subunit I	Head: 25-1596<br>Body: 104-1675	Head: 1572<br>Body: 1572
	LNR_{cox1}	Long non-coding RNA		Head: 1403<br>Body: 1974
trnY-cox2	*trnY*	Tyrosine tRNA	Head: 61-133<br>Body: 139-211	Head: 73<br>Body: 73
	cox2	Cytochrome c oxidase subunit II	Head: 104-817<br>Body: 182-892	Head: 714<br>Body: 711
	$LNR_{trnY\text{-}cox2}$	Long non-coding RNA		Body: 2278
cox3-trnA	*cox3*	Cytochrome c oxidase subunit III	Head: 61-870<br>Body: 140-949	Head: 810<br>Body: 810
	trnA	Alanine tRNA	Head: 870-935<br>Body: 949-1014	Head: 66<br>Body: 66
	$LNR_{cox3\text{-}trnA}$	Long non-coding RNA		Body: 2137
nad1-trnQ	*nad1*	NADH subunit 1	Head: 1024-155<br>Body: 518-1387	Head: 870<br>Body: 870
	trnQ	Glutamine tRNA	Head: 115-59<br>Body: 1426-1484	Head: 57<br>Body: 59
	$LNR_{nad1\text{-}trnQ}$	Long non-coding RNA		Body: 2122
trnP-nad2-trnI	*trnP*	Proline tRNA	Head: 46-111<br>Body: 124-189	Head: 66<br>Body: 66
	nad2	NADH subunit 2	Head: 133-1010<br>Body: 211-1188	Head: 878<br>Body: 978
	trnI	Isoleucine tRNA	Head: 1108-1175<br>Body: 1188-1251	Head: 68<br>Body: 64
	$LNR_{trnP\text{-}nad2\text{-}trnI}$	Long non-coding RNA		Body: 1963
trnR-nad3	*trnR*	Arginine tRNA	Head: 45-111<br>Body: 165-229	Head: 67<br>Body: 65
	nad3	NADH subunit 3	Head: 111-473<br>Body: 230-592	Head: 363<br>Body: 363
	$LNR_{trnR\text{-}nad3}$	Long non-coding RNA		Body: 2546
trnK-nad4	*trnK*	Lysine tRNA	Head: 48-117<br>Body:170-239	Head: 70<br>Body: 70
	nad4	NADH subunit 4	Head: 118-1437<br>Body: 240-1559	Head: 1320<br>Body: 1320
	$LNR_{trnK\text{-}nad4}$	Long non-coding RNA		Head: 1903<br>Body: 1996
trnG-nad4L-trnV	*trnG*	Glycine tRNA	Head: 44-109	Head: 66

			Body: 122-185	Body: 64
	nad4L	NADH subunit 4L	Head: 115-384<br>Body: 186-461	Head: 270<br>Body: 276
	trnV	Valine tRNA	Head: 386-455<br>Body: 464-531	Head: 70<br>Body: 68
nad5	*nad5*	NADH subunit 5	Head: 25-1710<br>Body: 194-1789	Head: 1686<br>Body: 1686
	LNR_{nad5}	Long non-coding RNA		Body: 1867
trnF-nad6	*trnF*	Phenylalanine tRNA	Head: 68-138<br>Body: 127-197	Head: 71<br>Body: 71
	nad6	NADH subunit 6	Head: 122-646<br>Body: 180-705	Head: 525<br>Body: 525
$trnL_1$-*rrnS-trnC*	$trnL_1$	Leucine 1 (tag) tRNA	Head: 41-108<br>Body: 121-186	Head: 68<br>Body: 66
	rrnS	Small ribosomal subunit	Head: 163-902<br>Body: 242-981	Head: 740<br>Body: 740
	trnC	Cysteine tRNA	Head: 905-975<br>Body: 984-1054	Head: 71<br>Body: 71
	$LNR_{trnL1\text{-}rrnS\text{-}trnC}$	Long non-coding RNA		Body: 2196
$trnL_2$-*rrnL*	$trnL_2$	Leucine 2 (taa) tRNA	Head: 41-108<br>Body: 121-186	Head: 67<br>Body: 66
	rrnL	Large ribosomal subunit	Head: 162-1259<br>Body: 241-1336	Head: 1098<br>Body: 1096
	$LNR_{trnL2\text{-}rrnL}$	Long non-coding RNA		Body: 2067
$trnS_1$-*trnN-trnE*	$trnS_1$	Serine 1 (tct) tRNA	Head: 61-131<br>Body: 225-294	Head: 71<br>Body: 70
	trnN	Asparagine tRNA	Head: 157-223<br>Body: 320-386	Head: 67<br>Body: 67
	trnE	Glutamic acid tRNA	Head: 220-287<br>Body: 385-449	Head: 67<br>Body: 65
trnT-trnD-trnH	*trnT*	Threonine tRNA	Head: 88-115<br>Body: 100-167	Head: 68<br>Body: 68
	trnD	Aspartic acid tRNA	Head: 172-241<br>Body: 184-253	Head: 70<br>Body: 70
	trnH	Histidine tRNA	Head: 242-315<br>Body: 254-326	Head: 74<br>Body: 73
trnM	*trnM*	Methionine tRNA	Head: 64-130<br>Body: 126-190	Head: 67<br>Body: 65
trnW-$trnS_2$	*trnW*	Tryptophan tRNA	Head: 99-171<br>Body: 179-251	Head: 73<br>Body: 73
	$trnS_2$	Serine 2 (tga) tRNA	Head: 172-242<br>Body: 252-322	Head: 71<br>Body: 71

Note: [a] Head - human head louse, *Pediculus humanus capitis*; [b] Body - human body louse, *Pediculus humanus corporis*.
