# Supplementary material for: Transcription of the Extensively Fragmented Mitochondrial Genomes of Human Lice
Source: Biology (Basel). 2026 Feb 8;15(4):296. doi: 10.3390/biology15040296 (PMC12938707; doi:10.3390/biology15040296)
Supplement: Supplementary file 1 [file biology-15-00296-s001.zip › Supplementary Table S6.pdf]

Table S6: Normality tests of the transcription level data of mitochondrial genes, minichromosomes, motifs, coding regions, and non-coding regions of the human head louse, *Pediculus humanus capitis*, and the human body louse, *Pediculus humanus corporis*.

Coding and non-coding regions of mitochondrial minichromosomes

	Kolmogorov-Smirnov[a]			Shapiro-Wilk		
	Statistic	df	Sig.	Statistic	df	Sig.
Coding region (mean coverage)	.335	28	<.001	.505	28	<.001
Non-coding region (mean coverage)	.220	28	.001	.693	28	<.001

a. Lilliefors Significance Correction

atp8-atp6 motifs

	Kolmogorov-Smirnov[a]			Shapiro-Wilk		
	Statistic	df	Sig.	Statistic	df	Sig.
30 bp #2 (RPKM)	.255	8	.135	.811	8	.037
30 bp #1 (RPKM)	.240	8	.194	.825	8	.053
30 bp #3 (RPKM)	.253	8	.142	.856	8	.109

a. Lilliefors Significance Correction

AT-rich and GC-rich motifs in the long non-coding region of each mitochondrial minichromosome

	Kolmogorov-Smirnov[a]			Shapiro-Wilk		
	Statistic	df	Sig.	Statistic	df	Sig.
AT-rich motif (RPKM)	.194	22	.031	.802	22	<.001
50 bp upstream AT-rich motif (RPKM)	.235	22	.003	.683	22	<.001
GC-rich motif (RPKM)	.283	22	<.001	.786	22	<.001
50 bp downstream GC-rich motif (RPKM)	.301	22	<.001	.758	22	<.001

a. Lilliefors Significance Correction

Mitochondrial minichromosomes

	Kolmogorov-Smirnov[a]			Shapiro-Wilk		
	Statistic	df	Sig.	Statistic	df	Sig.
atp8-atp6 with NCS (RPKM)	.172	8	.200[*]	.908	8	.342
cob with NCS (RPKM)	.211	8	.200[*]	.886	8	.217
cox1 with NCS (RPKM)	.158	8	.200[*]	.908	8	.338
cox3-A with NCS (RPKM)	.159	8	.200[*]	.922	8	.444

F-nad6 with NCS (RPKM)	.226	8	.200*	.819	8	.046
G-nad4L-V with NCS (RPKM)	.201	8	.200*	.920	8	.433
K-nad4 with NCS (RPKM)	.209	8	.200*	.834	8	.065
L1-rrnS-C with NCS (RPKM)	.184	8	.200*	.950	8	.711
L2-rrnL with NCS (RPKM)	.262	8	.112	.866	8	.139
M with NCS (RPKM)	.276	8	.073	.819	8	.046
nad1-Q with NCS (RPKM)	.313	8	.020	.801	8	.029
nad5 with NCS (RPKM)	.177	8	.200*	.872	8	.159
P-nad2-I with NCS (RPKM)	.200	8	.200*	.915	8	.394
R-nad3 with NCS (RPKM)	.169	8	.200*	.945	8	.660
S1-N-E with NCS (RPKM)	.270	8	.087	.728	8	.005
T-D-H with NCS (RPKM)	.501	8	<.001	.444	8	<.001
W-S2 with NCS (RPKM)	.142	8	.200*	.938	8	.594
Y-cox2 with NCS (RPKM)	.150	8	.200*	.959	8	.799

*. This is a lower bound of the true significance

a. Lilliefors Significance Correction

Mitochondrial protein-coding and rRNA genes

	Kolmogorov-Smirnov[a]			Shapiro-Wilk		
	Statistic	df	Sig.	Statistic	df	Sig.
atp6 (RPKM)	.239	8	.200*	.826	8	.054
atp8 (RPKM)	.254	8	.137	.863	8	.127
cob (RPKM)	.227	8	.200*	.917	8	.407
cox1 (RPKM)	.307	8	.025	.805	8	.033
cox2 (RPKM)	.295	8	.039	.866	8	.137
cox3 (RPKM)	.356	8	.004	.711	8	.003
nad1 (RPKM)	.302	8	.030	.821	8	.047
nad2 (RPKM)	.201	8	.200*	.873	8	.162
nad3 (RPKM)	.221	8	.200*	.907	8	.331
nad4 (RPKM)	.230	8	.200*	.876	8	.174
nad4L (RPKM)	.321	8	.015	.777	8	.016
nad5 (RPKM)	.197	8	.200*	.937	8	.585
nad6 (RPKM)	.274	8	.078	.812	8	.039
rrnL (RPKM)	.202	8	.200*	.955	8	.759
rrnS (RPKM)	.169	8	.200*	.948	8	.691

*. This is a lower bound of the true significance

a. Lilliefors Significance Correction

Mitochondrial tRNA genes

	Kolmogorov-Smirnov[a]			Shapiro-Wilk		
	Statistic	df	Sig.	Statistic	df	Sig.
trnA (RPKM)	.264	8	.107	.809	8	.036
trnC (RPKM)	.255	8	.135	.862	8	.126
trnD (RPKM)	.258	8	.125	.817	8	.043
trnE (RPKM)	.312	8	.021	.719	8	.004
trnF (RPKM)	.300	8	.033	.816	8	.042
trnG (RPKM)	.355	8	.004	.599	8	<.001
trnH (RPKM)	.389	8	<.001	.687	8	.002
trnI (RPKM)	.314	8	.019	.778	8	.017
trnK (RPKM)	.195	8	.200*	.867	8	.142
trnL1 (RPKM)	.175	8	.200*	.900	8	.288
trnL2 (RPKM)	.334	8	.009	.745	8	.007
trnM (RPKM)	.226	8	.200*	.871	8	.154
trnN (RPKM)	.313	8	.020	.718	8	.004
trnP (RPKM)	.158	8	.200*	.939	8	.601
trnQ (RPKM)	.256	8	.130	.817	8	.043
trnR (RPKM)	.224	8	.200*	.827	8	.056
trnS1 (RPKM)	.313	8	.020	.718	8	.004
trnS2 (RPKM)	.332	8	.010	.738	8	.006
trnT (RPKM)	.211	8	.200*	.871	8	.154
trnV (RPKM)	.174	8	.200*	.965	8	.860
trnW (RPKM)	.339	8	.007	.714	8	.003
trnY (RPKM)	.262	8	.113	.807	8	.034

*. This is a lower bound of the true significance

a. Lilliefors Significance Correction

Mitochondrial minichromosomes including non-coding sequences (NCS)

	Kolmogorov-Smirnov[a]			Shapiro-Wilk		
	Statistic	df	Sig.	Statistic	df	Sig.
atp8-atp6 with NCS (TPM)	.199	8	.200*	.906	8	.324
cob with NCS (TPM)	.216	8	.200*	.946	8	.673
cox1 with NCS (TPM)	.187	8	.200*	.914	8	.384
cox3-A with NCS (TPM)	.268	8	.095	.835	8	.066
F-nad6 with NCS (TPM)	.308	8	.025	.777	8	.016
G-nad4L-V with NCS (TPM)	.193	8	.200*	.939	8	.603

K-nad4 with NCS (TPM)	.242	8	.185	.929	8	.504
L1-rrnS-C with NCS (TPM)	.136	8	.200*	.963	8	.840
L2-rrnL with NCS (TPM)	.245	8	.171	.819	8	.045
M with NCS (TPM)	.282	8	.060	.794	8	.025
nad1-Q with NCS (TPM)	.257	8	.128	.870	8	.151
nad5 with NCS (TPM)	.221	8	.200*	.843	8	.081
P-nad2-I with NCS (TPM)	.195	8	.200*	.918	8	.416
R-nad3 with NCS (TPM)	.191	8	.200*	.920	8	.431
S1-N-E with NCS (TPM)	.282	8	.060	.740	8	.006
T-D-H with NCS (TPM)	.481	8	<.001	.456	8	<.001
W-S2 with NCS (TPM)	.148	8	.200*	.922	8	.448
Y-cox2 with NCS (TPM)	.229	8	.200*	.883	8	.203

*. This is a lower bound of the true significance

a. Lilliefors Significance Correction

Mitochondrial protein-coding and rRNA genes

	Kolmogorov-Smirnov[a]			Shapiro-Wilk		
	Statistic	df	Sig.	Statistic	df	Sig.
atp6 (TPM)	.227	8	.200*	.851	8	.096
atp8 (TPM)	.261	8	.115	.857	8	.113
cob (TPM)	.212	8	.200*	.913	8	.374
cox1 (TPM)	.229	8	.200*	.840	8	.075
cox2 (TPM)	.307	8	.025	.859	8	.117
cox3 (TPM)	.326	8	.013	.764	8	.012
nad1 (TPM)	.284	8	.057	.809	8	.036
nad2 (TPM)	.180	8	.200*	.888	8	.225
nad3 (TPM)	.258	8	.125	.893	8	.247
nad4 (TPM)	.287	8	.051	.900	8	.290
nad4L (TPM)	.313	8	.021	.770	8	.014
nad5 (TPM)	.199	8	.200*	.959	8	.802
nad6 (TPM)	.286	8	.053	.806	8	.033
rrnL (TPM)	.202	8	.200*	.950	8	.708
rrnS (TPM)	.129	8	.200*	.963	8	.834

*. This is a lower bound of the true significance

a. Lilliefors Significance Correction

Mitochondrial tRNA genes

	Kolmogorov-Smirnov[a]			Shapiro-Wilk		
	Statistic	df	Sig.	Statistic	df	Sig.
trnA (TPM)	.225	8	.200*	.849	8	.092
trnC (TPM)	.246	8	.170	.826	8	.055
trnD (TPM)	.302	8	.031	.795	8	.025
trnE (TPM)	.296	8	.038	.807	8	.034
trnF (TPM)	.320	8	.015	.801	8	.029
trnG (TPM)	.363	8	.003	.582	8	<.001
trnH (TPM)	.386	8	<.001	.639	8	<.001
trnI (TPM)	.301	8	.031	.801	8	.029
trnK (TPM)	.194	8	.200*	.858	8	.116
trnL1 (TPM)	.192	8	.200*	.901	8	.297
trnL2 (TPM)	.328	8	.011	.751	8	.008
trnM (TPM)	.242	8	.184	.827	8	.055
trnN (TPM)	.297	8	.037	.806	8	.033
trnP (TPM)	.166	8	.200*	.931	8	.525
trnQ (TPM)	.263	8	.109	.832	8	.062
trnR (TPM)	.237	8	.200*	.809	8	.036
trnS1 (TPM)	.296	8	.037	.806	8	.033
trnS2 (TPM)	.331	8	.010	.753	8	.009
trnT (TPM)	.211	8	.200*	.872	8	.156
trnV (TPM)	.206	8	.200*	.957	8	.778
trnW (TPM)	.342	8	.006	.725	8	.004
trnY (TPM)	.238	8	.200*	.846	8	.086

*. This is a lower bound of the true significance

a. Lilliefors Significance Correction
