# Supplementary material for: Transcription of the Extensively Fragmented Mitochondrial Genomes of Human Lice
Source: Biology (Basel). 2026 Feb 8;15(4):296. doi: 10.3390/biology15040296 (PMC12938707; doi:10.3390/biology15040296)
Supplement: Supplementary file 1 [file biology-15-00296-s001.zip › Supplementary Table S8.pdf]

Table S8: Comparison between the coding and non-coding region of each mitochondrial minichromosome of the human body louse, *Pediculus humanus corporis*, using the Wilcoxon signed-ranks test.

Ranks

		N	Mean Rank	Sum of Ranks
Non-Coding Coverage – Coding Coverage	Negative Ranks	22[a]	11.50	253.00
	Positive Ranks	0[b]	0.00	0.00
	Ties	0[c]		
	Total	22		

a. Non-Coding Coverage < Coding Coverage

b. Non-Coding Coverage > Coding Coverage

c. Non-Coding Coverage = Coding Coverage

Test Statistics[d]

	Non-Coding Coverage – Coding Coverage
Z	-4.107
Asymp. Sig. (2-tailed)	0.00004

d. Wilcoxon Signed Ranks Test

e. Based on positive ranks.
